# Nitric oxide modulates interleukin-2-induced proliferation in CTLL-2 cells

**DOI:** 10.1155/S0962935196000464

**Published:** 1996-10

**Authors:** J. Padrón, L. Glaría, O. Martinez, M. Torres, E. Lopez, R. Delgado, L. Caveda, A. Rojas

**Affiliations:** 1 Finlay Institute Department of Basic and Clinical Immunology P.O. Box 16017 Havana Cuba; 2 Center of Pharmaceutical Chemistry Department of Pharmacology and Toxicology P.O. Box 6990 Havana Cuba

## Abstract

The role of the L-arginine–nitric oxide metabolic pathway was explored for interleukin-2-induced proliferation in the cytotoxic T lymphocyte clone CTLL-2. Specific inhibition of nitric oxide synthase significantly diminished, in a concentration-dependent manner, ^3^H-thymidine uptake of CTLL-2 cells in response to different concentrations of interleukin 2. Withdrawal of L-arginine from culture medium resulted as potent as the higher inhibition obtained when blocking nitric oxide synthase with L-arginine analogues. Furthermore, intermedial concentrations of Larginine and exogenous nitric oxide donors were found for achieving optimal IL2-induced proliferation of CTLL-2. These findings prompted us to suggest that intra- and/or inter-cellular nitric oxide signalling may contribute to the modulation of the IL2 mitogenic effect upon cytotoxic T lymphocytes.


Mediators of Inflammation, 5, 324–327 (1996)

### Nitric oxide modulates interleukin-2-induced proliferation in CTLL-2 cells

J. Padrón,[1,CA] L. Glaría,[2] O. Martínez,[2] M. Torres,[2] E. Lopez,[2] R. Delgado,[2] L. Caveda[2] and A. Rojas[2]

[1]Finlay Institute, Department of Basic and Clinical Immunology, P.O. Box 16017, Havana, Cuba; and [2]Center of Pharmaceutical Chemistry, Department of Pharmacology and Toxicology, P.O. Box 6990, Havana, Cuba

[CA]Corresponding Author
Fax: (+53) 7 336471 or 336075
Email: finlayci@ceniai.cu

**The role of the L-arginine–nitric oxide metabolic pathway was explored for interleukin-2-induced proliferation in the cytotoxic T lymphocyte clone CTLL-2. Specific inhibition of nitric oxide synthase significantly diminished, in a concentration-dependent manner, $^3$H-thymidine uptake of CTLL-2 cells in response to different concentrations of interleukin 2. Withdrawal of L-arginine from culture medium resulted as potent as the higher inhibition obtained when blocking nitric oxide synthase with L-arginine analogues. Furthermore, intermedial concentrations of L-arginine and exogenous nitric oxide donors were found for achieving optimal IL2-induced proliferation of CTLL-2. These findings prompted us to suggest that intra- and/or inter-cellular nitric oxide signalling may contribute to the modulation of the IL2 mitogenic effect upon cytotoxic T lymphocytes.**

**Key words:** CTLL-2, Immunoregulation, Interleukin-2, Nitric oxide, Proliferation

### Introduction

Interleukin-2 (IL2) is among the principal lymphokines used in humans, either alone or in combination with lymphokine-activated killer cells, to treat advanced cancer.[1] Its therapeutic proficiency has been related with the clonal expansion of a broad range of T cell types, but particularly cytotoxic T lymphocytes.[2] However, despite many efforts, most of the signal transduction pathways involved in the IL2-induced proliferation are yet to be elucidated.

Meanwhile, the induction of high nitric oxide (NO) levels has been suggested as being responsible for some of the adverse side effects of IL2 treatment,[3] and further findings have demonstrated the beneficial effect of concomitant administration of L-arginine for improving IL2-induced immune stimulation.[4]

Two major nitric oxide synthases (NOS) have been reported; the inducible (iNOS) mainly dependent on inflammatory stimuli[5] and the constitutive (cNOS) controlled by calmodulin and free $Ca^{2+}$ concentrations.[6,7] Both enzymes are, in fact, capable of using L-arginine and molecular oxygen as substrates for producing L-citrulline and NO,[8] however, the distinguishable characteristics of cNOS kinetic[9] let it function as an ubiquitous system for mediating the generation of cGMP,[7] whereas iNOS is principally associated with high output of NO for killing invaders and/or injuring affected tissues.

In this study we wanted to explore the role of different levels of endogenous or exogenous NO in the IL2-induced proliferation in a well-known clone of cytotoxic T lymphocytes, the CTLL-2 cell.

### Materials and Methods

LNMMA (Wellcome Research Labs) and sodium nitroprussiate (SNP) (Sigma) were freshly prepared at (2 mM) in corresponding culture medium and filtered through a 0.2 μm syringe filter (Millipore), and finally added in volumes of 50 μl/well to final concentrations of 500, 50 or 5 μM and 500, 50, 5, 0.5, 0.05, 0.005 or 0.0005 μM respectively.

The CTLL-2 (IL2-dependent subclone of cytotoxic T cells derived from a C57bl/6 mouse) were obtained from Dr M. Araña at the Center of Biological Research, Havana, Cuba. Cells were grown at 37°C and 5% of $CO_2$ in RPMI 1640 (ICN Flow) supplemented with streptomycin (100 μg/ml) (Gibco), penicillin (100 U/ml) (Gibco), L-glutamine (2 mM) (Gibco), 10% of heat-inactivated fetal calf serum (Gibco), 20% of T cell growth factor (Wellcome Research Labs) and 2 mercapto-ethanol (5 μM) (Sigma).

Prior to experimentation, cells were washed twice in fresh RPMI 1640 and later suspended ($1 \times 10^6$ cells/ml) in the same culture medium used for maintenance but without T cell growth factor. Cell suspension was then plated at

© 1996 Rapid Science Publishers

$1 \times 10^5$ cell/well and pretreated for 30 min with L-NMMA or SNP, and finally stimulated with IL2 (Dinatec) in volumes of 50 µl/well to final concentrations of 20, 10 or 5 U/ml.

In order to analyse the effect of L-arginine on the IL2-induced proliferation, cells were washed, plated and treated as described above, but in RPMI 1640 free from L-arginine (ICN Flow) instead of normal RPMI 1640. The supplement of L-arginine (ICN Flow) was adjusted to different concentrations by diluting in L-arginine free RPMI 1640, and finally added in volumes of 50 µl/well.

The proliferation of CTLL-2 was assumed to be a function of the counts per minute (cpm) of the DNA fraction after incubation for the last 8–12 h of culture with 1 µCi of $^3$H-thymidine (Amersham) (added in volumes of 25 µl/well). All plates were frozen until harvested and counted for 60 s on a liquid scintillation counter.

The measurement of nitrite was based on the reaction of equal volumes of sample and Griess reagent, which briefly consists of a mixture of α naphtyl amine at 0.1% in water and sulphanilamide at 1% in phosphoric acid at 5% (1:1 v/v). After 10 min light absorbance was measured at 540 nm.

All data are expressed as mean ± standard error from at least three experiments each with three replicates. Comparison between groups was based on Student's *t*-test and significant difference was accepted as $p \leq 0.05$.

### Results

A normal curve of CTLL-2 growth in the presence of different concentrations of IL2 was clearly evident after 24 h of culture (Fig. 1). Additionally, it was also observed that in all cases administration of the specific NOS antagonist LNMMA (500 µM) was capable of significantly diminishing $^3$H-thymidine uptake. Similar results were obtained in previous experiments when T cell growth factor was used instead of IL2 (data not shown).

Further results (Fig. 2) demonstrated that, in the presence of L-arginine (0.1 mM) and fixed concentration of IL2 (20 U/ml), the specific NOS antagonist LNMMA showed a concentration-dependent inhibitory effect upon CTLL-2 growth. However, in the absence of L-arginine, $^3$H-thymidine uptake in all cases remained at a basal plateau resembling that observed when treated with LNMMA (500 µM).

Consistently (Fig. 3), when different concentrations of L-arginine were assayed for CTLL-2 $^3$H-thymidine uptake in the presence of fixed concentration of IL2 (5 U/ml), it was noted that there was maximal proliferation at around 0.3 mM. Either very low or very high levels of L-arginine had significantly lower values of $^3$H-thymidine uptake.

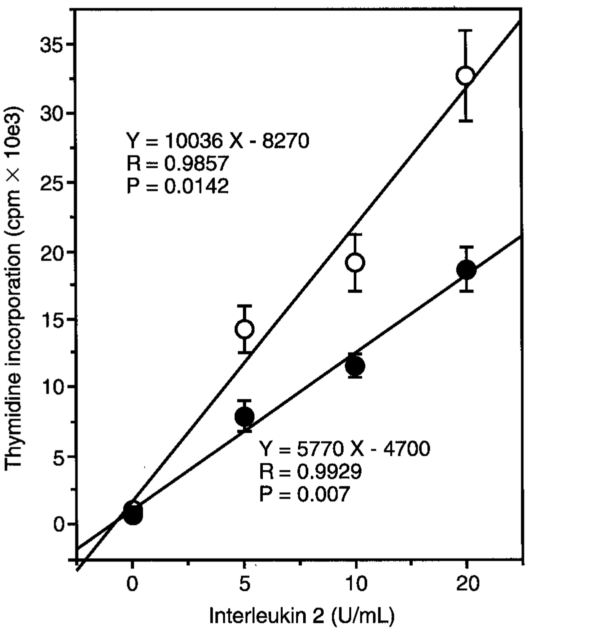

FIG. 1. Proliferation profile of CTLL-2 cells after 24 h of cultures in responses to different concentrations of IL2, in absence (open circles) or presence (solid circles) of LNMMA at 500 µM. Each point is the mean ± SEM of three experiments. Equation of the linear regression curve is also expressed on each case.

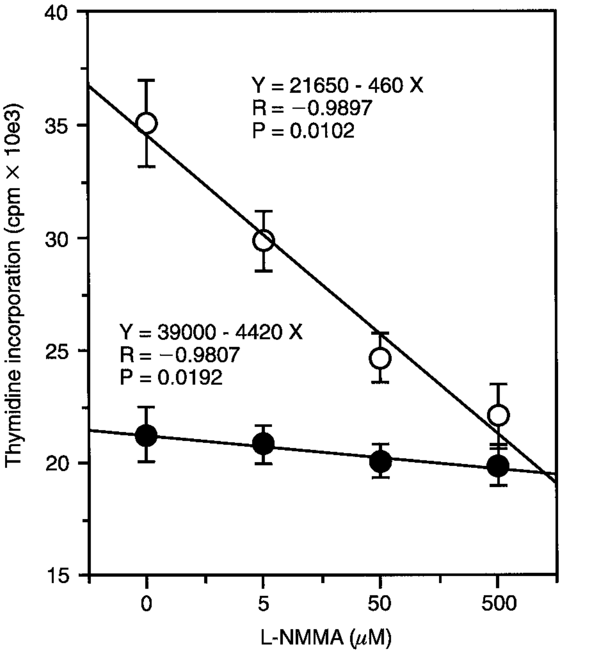

FIG. 2. Concentration-dependent inhibitory effect of L-NMMA upon IL2 (20 U/ml)-induced proliferation on CTLL-2 cells, in presence (2 mM) (open circles) or absence (solid circles) of L-arginine. The absence of L-arginine was as potent as L-NMMA (500 µM) for reducing CTLL-2 cells proliferation. Each point is the mean ± SEM of three experiments. Equation of the linear regression curve is also expressed on each case.

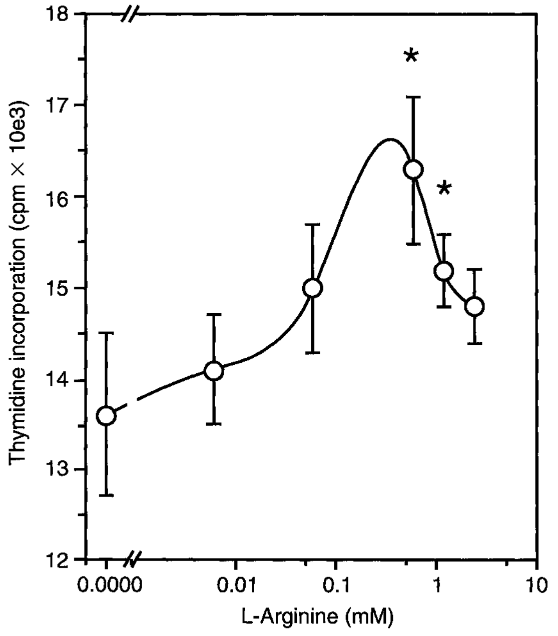

FIG. 3. Concentration-dependent modulatory effect of L-arginine upon the proliferation induced by IL2 (5 U/ml) on CTLL-2 cells. Each point is the mean ± SEM of three experiments. Student's *t*-test was assessed *vs* baseline. Significant difference was accepted for * $p < 0.05$.

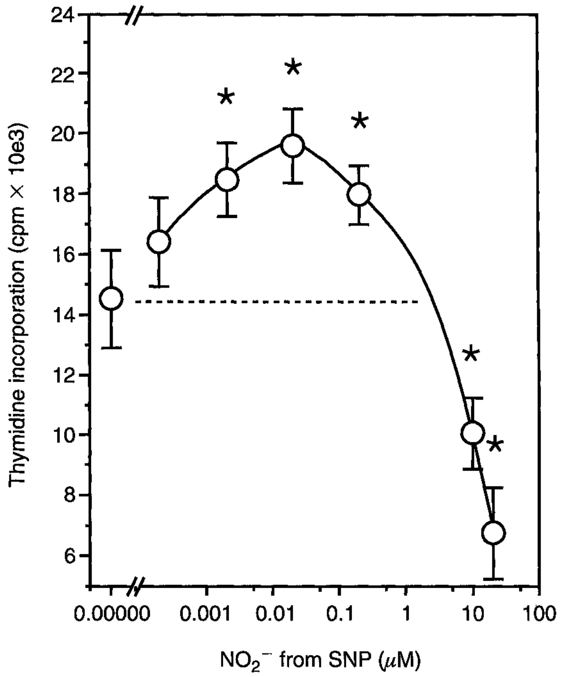

FIG. 4. Concentration-dependent modulatory effect of the SNP-derived NO upon proliferation of CTLL-2 cells in response to IL2 (5 U/ml). The stable metabolite nitrite was measured as index of NO generation from SNP. Each point is the mean ± SEM of three experiments. Student's *t*-test was assessed *vs* baseline. Significant difference was accepted for * $p < 0.05$.

Finally, it was observed that even exogenous administration of NO was able to modulate the proliferation of CTLL-2 cells in response to IL2 (5 U/ml) (Fig. 4). A well-known NO generator (SNP) was added in a wide range of concentrations and, as with L-arginine, there seems to exist a critical value of concentration of NO (around 20 pmol/ml) for which the proliferation was maximal. Either lower or higher levels of NO may affect the optimal proliferation of CTLL-2 cells.

### Discussion

The involvement of NO in the control of the mitogenic activation of T cells was firstly described in models of whole spleen cells proliferation in response to Con A.[10] In that case it was assumed that the NO coming from the co-cultured activated macrophages might be functioning as a suppressor mechanism to downregulate T cells growth. Further studies, however, reported the finding that the NO metabolic pathway was essential for the normal DNA synthesis in purified lymphocytes growing in response to Con A.[11] Such apparent contradiction could be basically explained because of the level and localization of NO generation, but it may be important to also consider the T cell type.

Indeed, different patterns of production and susceptibility to NO for two clones of T helper type 1 (Th1) and T helper type 2 (Th2) lymphocytes have been described.[12] The first seem to be potent generators of NO but at the same time they are susceptible to diminishing their own cytokine production and growth when high NO levels occur, meanwhile the second do not produce and remain apparently unaffected to high NO levels.

Curiously CTLL-2 cells in our system failed to generate high NO levels as a final effector molecule after IL2 activation (data not shown). It might be thought that NO is actually not important for their cytotoxicity or for the full activation of CTLL-2 further stimuli are required, such as close contact with target cells or combination with other cytokines or humoral factors.

In any case it is interesting that CTLL-2 cells may be using its production of low levels of NO[13] as an intracellular signal transduction pathway for the IL2-induced proliferation, without excluding that, when high NO level occurs, a typical negative feed-back mechanism downregulate their own growth. It should be important to note that exogenous NO may also modulate the IL2-induced proliferation on CTLL-2, suggesting that even the NO generated by surrounding cells might be actively contributing to the modulation of cytotoxic T lymphocytes proliferation.

Considering that for macrophages such dependence on low levels of NO for cGMP generation during activation is also probable[14] meanwhile high NO levels mediate its own programmed cell death,[15] it is possible to speculate that the NO metabolic pathway is not only differentially produced as a final effector molecule from a wide range of immune cells, but might also represent an additional humoral factor (beside cytokines) for modulating intercellular induced activation, growth and death.

The present report gives new evidence of the roles of the L-arginine-NO metabolic pathway for understanding the pharmacodynamics of IL2 therapy. Appropriately low levels of NO may guarantee clonal expansion of cytotoxic T lymphocytes encouraged for killing infected or transformed cells, but high NO levels may also inhibit mitogenic effects of IL2. Thus, an improvement on IL2 therapy might be possible by controlling the appropriate levels of L-arginine and/or NO. In that sense, new NO scavengers and/or novel isoform specific NOS inhibitors may represent valuable approaches.

### References

1. West WH, Tauer KW, Vannelli JR, *et al.* Constant infusion of recombinant interleukin-2 in adoptive immunotherapy of advanced cancer. *N Engl J Med* 1987; **316**: 898–904.
2. Hirshaut-Y, Slovin-SF. Harnessing T-lymphocytes for human cancer immunotherapy. *Cancer* 1985; **56**: 1366–1373.
3. Hibbs, JB, Westenfelder C, Taintor R, *et al.* Evidence for cytokine-inducible nitric oxide synthesis from L-arginine in patients receiving interleukin-2 therapy. *J Clin Invest* 1992; **89**: 867–877.
4. Leberman MD, Nishioka K, Redmon HP, Daly JM. Enhancement of interleukin-2 immunotherapy with L-arginine. *Ann Surg* 1992; **215**: 157–165.
5. Stuehr DJ, Cho HJ, Kwon NS, Weise M, Nathan CF. Purification and characterization of the cytokine-induced macrophage nitric oxide synthase: an FAD- and FMN-containing flavoprotein. *Proc Natl Acad Sci USA* 1991; **88**: 7773–7777.
6. Bredt DS, Snyder SH. Isolation of nitric oxide synthase, a calmodulin requiring enzyme. *Proc Natl Acad Sci USA* 1990; **87**: 682–685.
7. Knowles R, Palacios M, Palmer R, Moncada S. Formation of nitric oxide from L-arginine in the central nervous system: a transduction mechanism for stimulation of soluble guanylate cyclase. *Proc Natl Acad Sci USA* 1989; **86**: 5159–5162.
8. Leone A, Palmer R, Knowles R, Francis P, Ashton D, Moncada S. Constitutive and inducible nitric oxide synthase incorporate molecular oxygen into both nitric oxide and citrulline. *J Biol Chem* 1991; **266**: 23790–23795.
9. Knowles R, Palacios M, Palmer R, Moncada S. Kinetic characteristics of nitric synthase from rat brain. *Biochem J* 1990; **269**: 207–210.
10. Albina JE, Abate JA, Henry WL. Nitric oxide production is required for murine resident peritoneal macrophages to suppress mitogen-stimulated T cell proliferation. *J Immunol* 1991; **147**: 144–148.
11. Efron DT, Kirk SJ, Regan MC, Wasserkrug HL, Barbul A. Nitric oxide generation from L-arginine is required for optimal human peripheral blood lymphocyte DNA synthesis. *Surgery* 1991; **110**: 327–324.
12. Taylor-Robinson A, Liew F, Severn A, *et al.* Regulation of the immune response by NO differentially produced by T helper type 1 and T helper type 2 cells. *Eur J Immunol* 1994; **24**: 980–984.
13. Kirk SJ, Regan MC, Barbul A. Cloned murine T lymphocytes synthesize a molecule with the biological characteristics of nitric oxide. *Biochem Biophys Res Commun* 1990; **173**: 660–665.
14. Bromberg Y, Pigk E. Cyclic GMP metabolism in macrophages. *Cell Immunol* 1980; **52**: 73–83.9.
15. Sarih M, Souvannavong V, Adam A. Nitric oxide synthase induces macrophages death by apoptosis. *Biochem Biophys Res Commun* 1993; **191**: 503–508.

**Received 11 June 1996; accepted 16 August 1996**

## References

[PDF_00355] Bredt D. S., Snyder S. H. (1990). Isolation of nitric oxide synthetase, a calmodulin-requiring enzyme.. Proc Natl Acad Sci U S A.

[PDF_00379] Bromberg Y., Pick E. (1980). Cyclic GMP metabolism in macrophages. I. Regulation of cyclic GMP levels by calcium and stimulation of cyclic GMP synthesis by NO-generating agents.. Cell Immunol.

[PDF_00370] Efron D. T., Kirk S. J., Regan M. C., Wasserkrug H. L., Barbul A. (1991). Nitric oxide generation from L-arginine is required for optimal human peripheral blood lymphocyte DNA synthesis.. Surgery.

[PDF_00345] Hibbs J. B., Westenfelder C., Taintor R., Vavrin Z., Kablitz C., Baranowski R. L., Ward J. H., Menlove R. L., McMurry M. P., Kushner J. P. (1992). Evidence for cytokine-inducible nitric oxide synthesis from L-arginine in patients receiving interleukin-2 therapy.. J Clin Invest.

[PDF_00343] Hirshaut Y., Slovin S. F. (1985). Harnessing T-lymphocytes for human cancer immunotherapy.. Cancer.

[PDF_00376] Kirk S. J., Regan M. C., Barbul A. (1990). Cloned murine T lymphocytes synthesize a molecule with the biological characteristics of nitric oxide.. Biochem Biophys Res Commun.

[PDF_00357] Knowles R. G., Palacios M., Palmer R. M., Moncada S. (1989). Formation of nitric oxide from L-arginine in the central nervous system: a transduction mechanism for stimulation of the soluble guanylate cyclase.. Proc Natl Acad Sci U S A.

[PDF_00365] Knowles R. G., Palacios M., Palmer R. M., Moncada S. (1990). Kinetic characteristics of nitric oxide synthase from rat brain.. Biochem J.

[PDF_00361] Leone A. M., Palmer R. M., Knowles R. G., Francis P. L., Ashton D. S., Moncada S. (1991). Constitutive and inducible nitric oxide synthases incorporate molecular oxygen into both nitric oxide and citrulline.. J Biol Chem.

[PDF_00348] Lieberman M. D., Nishioka K., Redmond H. P., Daly J. M. (1992). Enhancement of interleukin-2 immunotherapy with L-arginine.. Ann Surg.

[PDF_00381] Sarih M., Souvannavong V., Adam A. (1993). Nitric oxide synthase induces macrophage death by apoptosis.. Biochem Biophys Res Commun.

[PDF_00351] Stuehr D. J., Cho H. J., Kwon N. S., Weise M. F., Nathan C. F. (1991). Purification and characterization of the cytokine-induced macrophage nitric oxide synthase: an FAD- and FMN-containing flavoprotein.. Proc Natl Acad Sci U S A.

[PDF_00373] Taylor-Robinson A. W., Liew F. Y., Severn A., Xu D., McSorley S. J., Garside P., Padron J., Phillips R. S. (1994). Regulation of the immune response by nitric oxide differentially produced by T helper type 1 and T helper type 2 cells.. Eur J Immunol.

[PDF_00340] West W. H., Tauer K. W., Yannelli J. R., Marshall G. D., Orr D. W., Thurman G. B., Oldham R. K. (1987). Constant-infusion recombinant interleukin-2 in adoptive immunotherapy of advanced cancer.. N Engl J Med.

